# Reflecting on Dunbar’s numbers: Individual differences in energy allocation to personal relationships

**DOI:** 10.1371/journal.pone.0319604

**Published:** 2025-03-11

**Authors:** Wenbo Li, David S. Lee, Jonathan L. Stahl, Joseph Bayer

**Affiliations:** 1 School of Communication and Journalism, Stony Brook University, Stony Brook, New York, USA; 2 Department of Communication, University at Buffalo, Buffalo, New York, USA; 3 Department of Psychology, The Ohio State University, Columbus, Ohio, USA; 4 School of Communication, The Ohio State University, Columbus, Ohio, USA; 5 Translational Data Analytics Institute, The Ohio State University, Columbus, Ohio, USA; University of Glasgow, UNITED KINGDOM OF GREAT BRITAIN AND NORTHERN IRELAND

## Abstract

Past studies have investigated the variability in how people engage with their personal networks, yet less is known about how people *perceive* their energy allocation to different ties. Drawing on an online survey sample (N =  906), we tested whether subjective perceptions of energy allocation conform to so-called Dunbar’s Number(s). In addition, we evaluated the predictive roles of Big Five personality traits and self-esteem while controlling for differences in network structure. Results revealed significant heterogeneity in perceived energy allocation to different layers of personal networks (i.e., inner 5 vs. middle 15 vs. outer 150 relationships). In contrast to expectations, extraversion was not associated with perceived energy allocation, whereas self-esteem was associated with greater energy allocation to the middle (vs. inner) network layer. Our findings add to our knowledge of how people perceive relationship maintenance *across* their personal networks, along with the links to key psychological traits. More broadly, the findings suggest that more attention should be paid to psychological implications of the middle layer of personal networks. To conclude, we discuss the importance of studying individual differences in how people prioritize – and reflect on – different relationships in their networks.

## Introduction

Cultivating supportive relationships with a diverse set of individuals is critical to well-being and health [1–6]. To maintain and manage these relationships, however, individuals must invest considerable time and effort [7,8]. Because people have limited time and energy [9,10], how they decide to allocate their resources to different relationships—i.e., energy allocation—is critical to relationship maintenance and development [11]. And this process is only becoming more significant with the aid of online technologies. Indeed, key perspectives suggest that common tools for interacting today (e.g., social media feeds, messaging groups) allow people to monitor and manage their relationships with unprecedented ease and flexibility [11–15]. This possibility poses interesting questions for researchers as well as the lay public: How do people differ in the amount of time and effort they exert to maintain different relationships? What psychological factors underlie these individual differences in energy allocation?

### Energy allocation and Dunbar’s number

Scholars across the social sciences have long sought to understand how personal networks are developed and maintained. One pivotal framework for understanding the nature of human social networks comes from Dunbar and colleagues, which involves two interconnected theoretical perspectives: the social brain hypothesis [16] and Dunbar’s Number [17]. The social brain hypothesis posits that primates (including humans) evolved large brains to support their complex social lives. Earlier work revealed a surprisingly consistent positive relationship between the size of the brain—specifically, the neocortex—and the size of social groups [16,18]. The ability to process the complexities of social environments is constrained by a number of factors, including time available for interaction [10,18], memory capacity [9], social motivations [19], and cognitive abilities [20]. Due to these constraints, the social brain hypothesis argues, humans have developed relatively large brains to process and manipulate information about the social world. Hence, the social brain hypothesis centers on the cognitive constraints of developing and maintaining relationships—in particular, how many meaningful relationships an individual can have on average.

Dunbar and colleagues have thus sought to quantify the precise number of social relationships an individual maintains in their daily lives. More precisely, Dunbar has provided evidence that human social networks on average have approximately 150 relationships [21]. The number 150, in turn, has come to be known as “Dunbar’s Number” [17,22]. Dunbar’s Number has also been shown to hold in online settings (under certain assumptions). For instance, Gonçalves et al. [23] analyzed 1.7 million Twitter users’ conversation data across six months and found that these users had a maximum of 100–200 stable social relationships on Twitter, which is generally consistent with Dunbar’s Number. Similar results were found on large-scale mobile phone data [24] and email data [25], lending added support to Dunbar’s Number across several online mediums [26–28].

Of course, the 150 relationships (on average) in individuals’ networks are not homogenous. Based on emotional closeness, Dunbar identified approximately three layers of human social relationships (also referred to as a *Dunbar graph*; [29,30]). These 150 relationships can be organized into three hierarchically inclusive layers of increasing size but decreasing emotional intensity [31]. The innermost layer is defined as the “support clique” which contains individuals who provide significant social support in times of need (e.g., best friends). This group has a mean size of five [32]. The next layer is the “sympathy group” which includes those are relatively close, but not the closest—akin to “good friends”. This middle layer has a mean size of 10, inclusive of the inner layer support group [9,32]. Of note, the two inner layers (top 15 relationships combined), map on to what is commonly referred to as “core networks” [33,34]. Finally, the outer layer contains mostly “weak ties”, as defined by [35], that can be seen as arm’s-length relationships or acquaintances. This focal layer has a mean size of 135 with individuals who are viewed as less emotionally close and contacted less often [36].

### Individual differences in energy allocation

Although “Dunbar’s numbers” are helpful for understanding key relational thresholds of social networks, their work does not directly explain the level of heterogeneity in how people allocate their energy across different relationships (cf. [24]). To be sure, follow-up research has also recognized that there is wide variance around the mean sizes of Dunbar’s network layers [28]. Moreover, other researchers have argued that network size may be larger in online contexts, thus questioning the numerical limits enumerated above (e.g., [13,14]). Such concerns follow research revealing significant variations in personal network size (e.g., [28,37]), as well as structure ) and perception (e.g., [38,39]). These lines of work not only show that people differ in how they construct and interact with their networks, but also suggest that their perceptions about how they allocate social energy are likely to vary considerably.

Why would individuals differ in their energy allocation to specific social relationships? Research has long demonstrated that people spend more time with those whom they like [40] and to whom they are more committed [41,42]. In other cases, people may spend more time with others simply because they are (physically) nearby. Studies have shown that people tend to seek support from those who are more available to them [43]. Other times, the decision to approach and interact with others may be driven by one’s needs (e.g., [43,44]). For instance, instrumental (vs. non-instrumental) others—those who can facilitate the attainment of a personal goal—are brought to mind more easily and approached more quickly [45,46]. Similarly, past work [47] has shown that people’s willingness to interact with others varied to the extent that these individuals could fulfill their goals (e.g., problem-solving vs. reducing stress). Thus, people may allocate more of their energy to specific relationships that fulfill more diverse needs—ranging from emotional support to informational resources.

Cumulatively, the decisions people make about which relationships to invest in reflect their overall energy allocation across their personal networks. While Dunbar and colleagues’ work solidifies the notion that people interact the most with a small subset of ties [16], past research overlooks how people perceive their energy distribution across their entire network. Indeed, our understanding of how individuals *think about* their energy allocation to relationships—and how they see their allocation across network layers—remains limited. Thus, the current study examined whether individuals exhibit significant differences in the extent to which they prioritize those inner ties. For example, one person may allocate 45% of their total energy to their five closest friends—i.e., the support clique—whereas another person may allocate 15% to the same number of relationships. Following Dunbar’s Numbers, we approached this question directly by asking individuals to reflect on how they devote energy across their network layers.

***RQ1a:*** *To what extent do people vary in how they allocate energy to the inner, middle, and outer layers of their personal networks?*

The idea that there should be variability in energy allocation to different parts of personal networks may also apply to the specific relationships *within* core networks. Core networks encompass the innermost and middle circles as identified in Dunbar’s number. Several theoretical perspectives provide reasons to expect that even among close relationships, there may exist differences in the amount of energy distributed to specific relationships. According to the hedonic flexibility principle [48], affective states (e.g., happiness) can influence how people prioritize with whom they decide to interact (e.g., best friend vs. friends). Recent work on social exploration-exploitation also highlights several processes that influence people’s decision to allocate their resources to different relationships, and these processes may vary by individuals [49]. Similarly, attachment theory research has long documented the importance of attachment styles in guiding how individuals interact with close others (e.g., [50,51]; see [52]). While securely attached individuals feel comfortable interacting with close others, avoidantly attached individuals sometimes prefer to stay distant from close others and anxiously attached individuals often report wanting to be more intimate to close others. Thus, just as people may vary in how they allocate their resources across their broader networks, they may allocate their energy unevenly within their core networks (e.g., 15% of their social energy to their “best” friend, but another might allocate 5% to each of their three closest friends).

***RQ1b*** *: To what extent do people vary in how they allocate energy to the relationships within their core networks?.*

### Psychological predictors of energy allocation

Prior research has shown that individuals’ social networks differ widely from another in terms of structure (e.g., size, density), and this is partly the product of psychological factors such as personality traits [37,53,54]. Although there is a large literature dealing with the psychological predictors of personal network structure (for reviews, see [54–56]), less is known about the predictors of energy allocation. Hence, echoing past perspectives on individual differences in social network characteristics, here we focus on personality and self-esteem as potential antecedents for how people see their personal networks in different ways.

Personality traits capture stable patterns of thinking, feeling, and acting over time [57]. Personality factors have been shown to underlie sociality [53,58] and communication patterns [59,60]. Research has also found that key dimensions of personality, such as extraversion, agreeableness, and neuroticism, are associated with personal network characteristics—whether measured from objective indices of network structure or subjective perceptions (see [55] for a review). One robustly validated taxonomy is the Big Five personality model, which encompasses the traits of extraversion, openness, conscientiousness, agreeableness, and neuroticism [57,60]—and is commonly employed in the context of social network research.

Extraversion is perhaps the most studied personality trait within personal network research. It is characterized by a positive attitude toward and prioritization of social engagement and activities [61]. Compared to introverts, extraverts have better social skills [62] and are more motivated to seek out social experiences [63]. As a result, extraverts tend to have larger networks than do introverts [64,65]. Research by Dunbar and colleagues has further found that extraverts have larger social networks at each of the three network layers described above: support clique, sympathy group, and outer layer [66]. Concurrently, extraverts tend to distribute their time more widely across social relationships, with their average emotional closeness to network members being lower than introverts [66]. This suggests that extraverts should assign relatively less energy to ties in inner layers (and thus more energy to more distant layers), compared to introverts. However, it is less clear whether the other traits making up the Big Five (i.e., neuroticism, agreeableness, openness, conscientiousness) have direct implications for energy allocation. Previous research suggests that these traits vary significantly in their influence on how individuals perceive, value, and engage with social relationships in their social networks [67,68], which may lead to different patterns of energy allocation across different relationships. Accordingly, this study examines how personality is associated with perceived energy allocation to (1) the three layers of personal networks and (2) relationships within core networks.

***RQ2a****: Are extraversion, agreeableness, conscientiousness, neuroticism, and openness* associated *with differential energy allocation across personal network layers?*
***RQ2b***: *Are extraversion, agreeableness, conscientiousness, neuroticism, and openness associated with differential energy allocation to personal relationships within core networks?*

Another potential driver of energy allocation perception is self-esteem. Self-esteem represents a person’s subjective judgment about their value [69]. Of particular relevance, sociometer theory argues that self-esteem acts as a psychological system to monitor the social environment and evaluate social relationships—i.e., a *sociometer* [70]. According to sociometer theory, people actively seek to increase their relational value and social acceptance, with self-esteem acting as a gauge of their effectiveness. Whereas people high in self-esteem feel that they are valued in the eyes of others, those low in self-esteem feel that they are not as valued by others. In other words, part of self-esteem reflects people’s *perceptions* about how much value other people place on them [71], requiring a global assessment of their social relations.

People develop a sense of self-esteem by gauging the attention received from others and the degree of acceptance and respect felt [72]. Reciprocally, people invest attention and energy back into their social relationships accordingly [73]. In this respect, self-esteem could reflect not just one’s overall perception of available social resources, but also the resources one expects to invest into personal relationships [74,75]. In this way, a sense of self-esteem may be derived most centrally from the relationships that are given the most time and energy in daily life. As of now, however, it is unclear whether there is a direct link between allocating more energy toward certain parts of one’s network and self-esteem. On the one hand, by concentrating one’s limited energy on the closest relationships, an individual may benefit from a supportive base in terms of their perceived social value [76–78]. On the other hand, a disproportional focus on the inner layer may also reflect the lack of broader social resources or confidence in maintaining less established relationships [56,79]. To address this gap in knowledge, we investigated the relationship between self-esteem and individual differences in energy allocation.

***RQ3a*** *: How is self-esteem associated with perceived energy allocation across personal network layers?.*
***RQ3b*** *: How is self-esteem associated with perceived energy allocation to relationships within core networks?*

## Method

The current study assessed *perceived* energy allocation by asking people how they wholistically distribute energy within their broader network and closest ties. Personal relationships are arranged in networked structures, and these social structures are arranged as cognitive structures in people’s minds [45,80]. As such, we adopted a fixed-size egocentric cognitive social structure (CSS, [81]) design given the potential of network dimensions to shape or constrain how people think about energy allocation [82]. The CSS approach is geared toward measuring how individuals see and think about their relationships (and, in this case, energy allocation to those relationships). At the same time, it is important to recognize that CSS is not just a method but also a framework that emphasizes understanding the perceptions and mental representations individuals hold about their personal networks [83,84]. Unlike studies that use social network analysis and rely on observable interactions or structural data, our study thus examined the subjective perceptions and cognitive processes that underlie how people navigate their social worlds. Taking a CSS approach allowed us to capture the nuanced ways individuals divide their energy across different ties, while also exploring how personality traits and self-esteem are associated with network perceptions. We also measured network density (i.e., how interconnected one’s relationships are) and communities (i.e., how many distinctive clusters of connections one has) to account for the role of structural factors. In this way, we attempt to isolate individual differences in perceived energy allocation from confounds such as the number or nature of relationships on participants’ minds.

### Participants

A total of 1065 participants provided complete data via Amazon Mechanical Turk (MTurk) in Spring, 2020 [recruitment period: April 29-30, 2020]. Consistent with prior research [82], we removed those who did not list 8 names as instructed in the name generator (n =  9) and those who listed indistinguishable names (n =  111), those who reported consecutive letters for the eight names in the name generator (n =  6), and those who reported different ages at the beginning and end of the questionnaire (as an attention check; n =  33). Additionally, we examined participants for potential straight lining by calculating the variance of responses across key variables and found no such cases in the data. A total of 906 participants were included in the final analysis. Participants were compensated $2.25 for their response to this online survey. The survey took an average of 17.48 minutes (*SD* =  8.90 minutes). The participants in the sample had a mean age of 36.43 years (*SD* =  10.72) with 59.94% identifying as female, 39.51% identifying as male, 0.55% selecting “other or prefer not to answer”. The Institutional Review Board at the corresponding author’s institution approved all aspects of this study prior to data collection.

### Procedure

Upon entering the study, the core idea behind Dunbar’s Number was explained to participants in lay terms, including an explanation of how individuals are limited in how many relationships they can maintain. After reading the explanation, participants completed a “name generator” questionnaire. The name generator approach is a commonly employed procedure for obtaining rich data about personal networks [85]. We used a fixed-size ego-centered CSS name generator design to measure individual differences in energy allocation in a controlled manner. By employing a fixed-size name generator, we held the number of relationships constant. In other words, this method results in each participant having the same number of relationships on their mind when reflecting on their personal networks. The fixed-size approach offers several benefits, putting key controls in place to minimize measurement biases. First, it provides homogeneous conditions to participants, obtaining standardized and comparable information about various types of social relationships [82,86]. Second, this approach allows for capturing the most salient relationships in participants’ minds without sacrificing the accuracy of recall [87]. Altogether, this approach allows for testing how people differ in encoding, characterizing, and retrieving information about their own personal networks without relying on participants to decide how many relationships are important enough to count.

Participants were asked to think about the 8 people in their life that they were closest to and live in their current city or town—that is, people they see face-to-face. Following past work (e.g., [82]), eight relationships were elicited to ensure an inclusive approach to measuring core networks—thus encompassing the five closest ties of the support clique—without overburdening participants. They were asked to type the first name of each relationship and ensure that all names were distinguishable (e.g., by including a distinct last initial). With the eight names (i.e., core networks) populated, participants were asked to think about how they engaged with these ties. Specifically, they were reminded that people differ in their distribution of energy toward relationships and asked to indicate how they divide their energy between each of the 8 relationships in percentages (adding up to 100%). Next, we collected information about key structural characteristics of participants’ networks, given the established linkages between network structure and perceptual processes [88].

**Fig 1:**
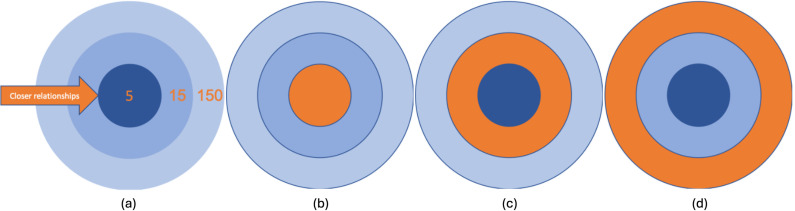
Visualizations of the three layers of Dunbar’s Number. (a) Overview of the three layers (b) The inner layer support group highlighted in orange (c) The middle layer empathy group highlighted in orange (d) The outer layer supported highlighted in orange.

After reporting on their core networks (top 8 relationships), participants were asked to think about their broader social network (top 150 relationships). A series of figures were provided to describe the differences between three circles within Dunbar’s framework: top 5 (Circle 1: the inner circle), top 15 (Circle 2: the middle circle), and top 150 (Circle 3: the outer circle). See Fig 1. Participants were then asked to report (in percentage) the amount of energy they divide across the three circles. Next, participants assessed their closeness to each of the 8 core network relationships and the closeness between each of the 8 individual pairs.

Following the network energy and closeness questions, participants answered questions from the Big Five Inventory (BFI-10) and the Single-Item Self Esteem Scale, and reported how satisfied they were with their overall social network (*network satisfaction*). At the end of the survey, participants answered questions about the type of their relationship (e.g., friend, coworker) with the nominated eight core network members. Finally, participants were asked about their demographic characteristics. See Online Supplemental Materials (OSM; https://osf.io/25zfb/ https://osf.io/25zfb/?view_only=52c78489a09442249831a5b9fd9ddee2) for a complete list of items.

### Measures

*Broader network allocation*. Following the work of Dunbar and colleagues, we first gave participants a brief description about Dunbar’s Number(s):

“*Research suggests that people can only maintain about 150 relationships at a time. Within these 150 people, relationships vary in their significance, and can be broken down into three circles. The first circle contains roughly 5 of your closest relationships. These are the people whom you talk to weekly and feel most supported by. The second circle contains the next 10 or so closest relationships (roughly 6*^*th*^ *to 15*^*th*^ *closest). These are the people whom you talk to monthly and would miss if you weren’t able to do so. The third circle contains your next 135 or so closest relationships (roughly 16*^*th*^ *to 150*^*th*^*). These are the people whom you don’t talk to regularly but would stop and have a conversation with if you bumped into them in daily life.*”

A series of figures were provided for each circle to facilitate a better understanding of Dunbar’s Number(s). After reading the explanation of the Dunbar’s Number(s), participants were asked “How do you divide your energy across the three circles in your social network” and reported energy allocation to each of the three circles of the Dunbar’s Number in percentage.

*Core network allocation*. Participants indicated the percentage of energy they allocated to each of the 8 relationships within their core networks (1^st^ relationship: *M* =  29.29%, *SD* =  19.05%; 2^nd^ relationship: *M* =  15.82%, *SD* =  8.33%; 3^rd^ relationship: *M* =  12.18%, *SD* =  6.74%; 4^th^ relationship: *M* =  9.99%, *SD* =  5.21%; 5^th^ relationship: *M* =  8.92%, *SD* =  6.16%; 6^th^ relationship: *M* =  8.42%, *SD* =  6.18%; 7^th^ relationship: *M* =  7.51%, *SD* =  5.49%; 8^th^ relationship: *M* =  7.89%, *SD* =  6.51%). The scores for 1^st^–5^th^ relationships were averaged for an index (*M* =  15.24%, *SD* =  2.72%) while the scores for the other 3 relationships (the 6^th^ to 8^th^ relationships) were averaged for an index (*M* =  7.93%, *SD* =  4.53%) to allow for more direct comparison with the Dunbar’s Circle 1 measure above.

**Fig 2:**
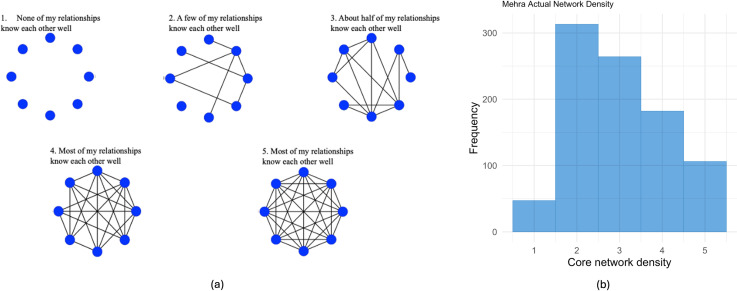
Visual scale [89] **(a) and histogram for core network density (b)**. Participants were given the following instructions: *The images below represent the connections between your current relationships. Each person is represented by a blue circle. Once again you are not pictured in the images. The more lines there are between people (circles), the more interconnected your relationships would be. Which of the choices below best represents how connected your relationships are to one another?.*

**Fig 3:**
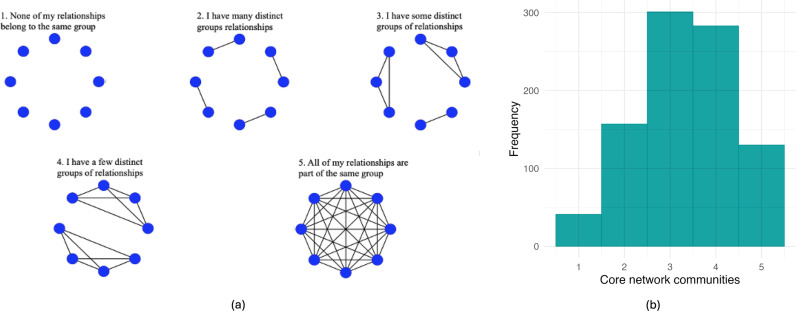
Visual scale [89] **(a) and histogram for core network communities (b).** Participants were given the following instructions: *Here, we once again want you to think about groups. Some of the relationships you entered may be part of the same groups—for example, you hang out together or are on the same trivia team. The images below represent the groups linked to your 8 relationships. Each group is represented by a set of connected circles. The more groups of people that are pictured (connected circles), the more distinct groups you belong to. Which of the choices below best represents the number of distinct groups that you belong to?*.

*Core network structure.* Participants were introduced to two ways to think about one’s social life: how interconnected the 8 relationships are (i.e., network density) and how many distinctive groups there are in the network consisting of the 8 relationships (i.e., network communities). They were then asked questions that assessed how they perceived their personal network density and communities. Self-reported network density was measured with a scale adapted from Mehra et al. ([89]; see Fig 2). Participants were asked how connected the eight nominated individuals in the name generator are to one another on a five-point visual network scale (1 =  *none of my relationships know each other well*; 5 =  *all of my relationships know each other well*). Self-reported network communities were measured with a scale adapted from Mehra et al. (2014; see Fig 3). Participants were asked how many distinct subgroups their core networks fall into on a five-point visual network scale (1 =  *none of my relationships belong to the same group*; 5 =  *all of my relationships are part of the same group*).

*Personality traits.* We measured personality traits using the BFI-10, an established scale for assessing the Big Five dimensions [90]. Participants were asked two questions on a 5-point scale (1 =  strongly disagree, 7 =  strongly agree) for each personality trait. Scores were summed for an index for each trait: extraversion (*M* =  8.12, *SD* =  2.64), agreeableness (*M* =  7.70, *SD* =  2.74); conscientious (*M* =  8.24, *SD* =  2.66); neuroticism (*M* =  7.76, *SD* =  2.49), and openness (*M* =  7.90, *SD* =  2.40).

*Self-esteem* was assessed using the Single-Item Self Esteem Scale (SISE) [91]. Participants rated the statement “I have high self-esteem” on a 5-point scale (1 =  not very true of me, 5 =  very true of me; *M* =  3.33, *SD* =  1.28).

*Network satisfaction* was measured by asking participants to rate the statement “I am satisfied with my social network” on a 5-point scale (1 =  not very true of me, 5 =  very true of me; *M* =  3.65, *SD* =  1.14).

*Socio-demographic factors*. Age, gender, and relationship status were measured and controlled in the data analysis given their potential to affect network structures and energy allocation to social relationships [36]. Research has shown that younger individuals tend to have larger networks, with men often having more contacts than women [92]; relationship status can also influence energy allocated to romantic partners versus broader social connections [93]. In addition, we collected the number of times participants had moved (*relocations;* 1 =  never, 6 =  five or more times; *M* =  2.20, *SD* =  1.67) to control for individual differences in hometown history. Frequent moves could impact network structure and energy allocation (particularly given the study procedure), as relocating creates opportunities to form new friendships, which require time and energy that might otherwise be spent maintaining existing ones [94].

### Analytic approach

RQ1 was addressed using descriptive statistics and visualizations. Our open entry data revealed considerable heterogeneity, and some extreme values, in the subjective ratings of perceived allocation energy across relationships. In turn, we ran parallel analyses that either included or excluded outliers to ensure the robustness of our results. The results did not differ significantly. Results including outliers are reported below (see Supplementary Materials for results excluding outliers). RQ2 and RQ3 were addressed using both ordinary least squares (OLS) regressions and rank regressions [95] to ensure that our results were robust to extreme values (without excluding valid observations). Specifically, we ran a separate regression model for each broader network layer to test RQ2a and RQ3a (top 5: Model 1a; top 6–15: Model 1b; top 16–150: Model 1c). In these models, energy allocation to the three network layers was regressed on Big Five personality traits and self-esteem. We ran another two regression models to test RQ2b and RQ3b for relationships within core networks (1^st^–5^th^: Model 2a; 6^th^–8^th^: Model 2b). In these models, energy allocation within core networks was regressed on Big Five traits and self-esteem. In addition to the covariates noted above, model 2a and 2b included covariates for the number of friends, family, and coworkers nominated (see [82]), along with  self-reported network density and communities. Missing values were listwise deleted. OLS and rank regression generated similar results. Below we report OLS regression results for all models, as OLS regression is generally more efficient in estimating coefficients. By minimizing the sum of squared residuals, it produces smaller standard errors and more precise estimates. Rank regression results can be found in the supplemental file on OSF https://osf.io/25zfb/?view_only=52c78489a09442249831a5b9fd9ddee2.

## Results

Table 1 presents the bivariate correlations between variables.

**Table 1:** Bivariate correlations between variables.

	1	2	3	4	5	6	7	8	9	10	11	12	13	14	15
1. Energy to top 5 in core network															
2. Energy to 6th‒8th in core network	‒1.00^**^														
3. Energy to Dunbar inner layer	.49^**^	‒.49^**^													
4. Energy to Dunbar middle layer	‒.29^**^	.29^**^	‒.71^**^												
5. Energy to Dunbar outer layer	‒.47^**^	.47^**^	‒.87^**^	.28^**^											
6. Calculated network density	‒.34^**^	.34^**^	‒.43^**^	.26^**^	.41^**^										
7. Calculated network communities	‒.12^**^	.12^**^	‒.03	.02	.03	.43^**^									
8. Self-reported network density	‒.13^**^	.13^**^	‒.19^**^	.13^**^	.16^**^	.45^**^	.15^**^								
9. Self-reported network communities	‒.09^*^	.09^*^	‒.18^**^	.16^**^	.13^**^	.37^**^	.18^**^	.50^**^							
10. Extraversion	.02	‒.02	.03	‒.03	‒.01	.00	.03	‒.00	.03						
11. Openness	.03	‒.03	.01	.01	‒.01	.05	.03	.03	.06	‒.01					
12. Conscientiousness	.04	‒.04	.07^*^	‒.08^*^	‒.04	.00	‒.04	‒.00	.03	‒.12^**^	‒.01				
13. Neuroticism	.01	‒.01	‒.03	.06	.00	‒.07^*^	‒.03	‒.01	.06	.06	.02	.10^**^			
14. Agreeableness	.06	‒.06	.01	‒.04	.01	.01	‒.05	‒.00	.01	‒.02	.01	.06	.03		
15. Self-esteem	‒.14^**^	.14^**^	‒.15^**^	.14^**^	.11^**^	.21^**^	.01	.10^**^	.12^**^	.01	.08^*^	.01	‒.05	‒.03	
16. Network satisfaction	‒.05	.05	‒.09^*^	.13^**^	.03	.19^**^	.06	.12^**^	.14^**^	.00	.05	‒.03	‒.07^*^	‒.02	.55^**^

*Note.*

* *p* < .05,

** *p* < .01

### Individual differences in energy allocation

**Fig 4:**
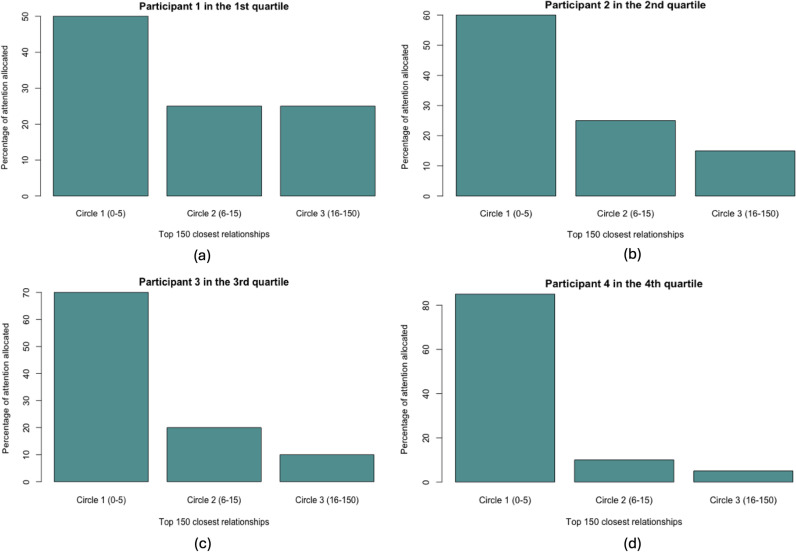
Variability in energy allocation to each layer of Dunbar’s Number indicated by four random participants in four quartiles (a, b, c, d) of the standard deviations (SDs) of energy allocation.

RQ1a asked to what extent people vary in how they allocate energy to relationships to the three layers of their personal networks (i.e., top 5, top 6–15, and top 16–150 relationships). Across the three Dunbar layers (top 5 relationships in the inner layer, top 6–15 relationships in the middle layer, and top 16–150 relationships in the outer layer), we found that participants allocated an average of 58.03% energy to the inner layer (*SD* =  20.49%), 25.44% to the middle layer (*SD* =  10.43%), and 16.53% to the outer layer (*SD* =  14.98%). Hence, participants’ perceptions of their energy allocation generally conformed to the Dunbar framework. We then computed the standard deviation (SD) of energy allocation for each participant to quantify the variability in energy distribution across participants. Fig 4 illustrates the wide individual differences in energy allocation across the three Dunbar layers by displaying one randomly selected participant from each of the four quartiles of energy allocation variability (SD).

**Fig 5:**
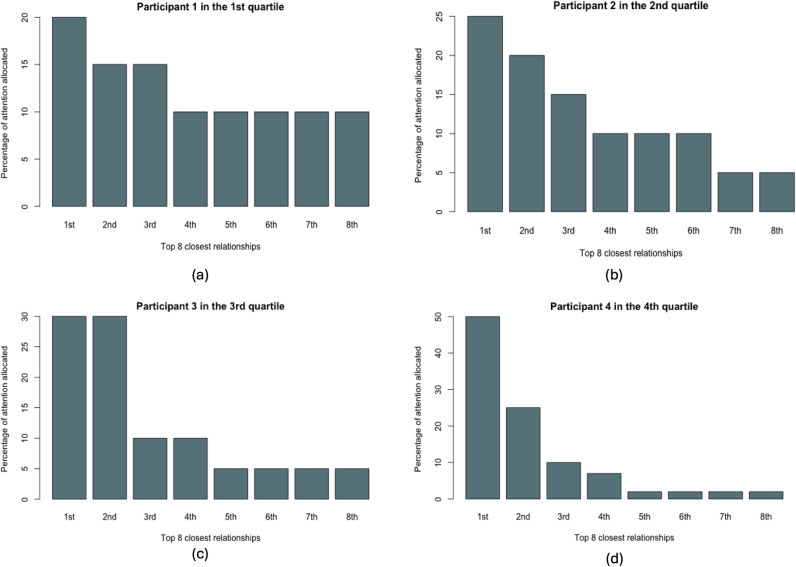
Variability in energy allocation to each relationship in core networks indicated by four random participants in four quartiles (a, b, c, d) of the standard deviations (SDs) of energy allocation.

RQ1b asked to what extent people vary in how they allocate energy to the relationships within their core networks (i.e., top 8 relationships). We found that participants allocated an average of 76.21% energy to the top 5 relationships (*SD* =  13.59%), which corresponds to the inner layer of Dunbar’s Numbers, and 23.79% to the 6^th^–8^th^ relationships (*SD* =  13.59%). Fig 5 visualizes the variability in energy allocation to the eight relationships in core networks. Taken together, our results indicated substantial variability in the perception of energy allocation to different components of their personal networks. Nonetheless, participants affirmed the centrality of Dunbar’s inner layers in terms of their relational attention.

We also examined the convergent validity of our broader and core network measures of energy allocation. Notably, energy allocation to the inner layer of the broader network was strongly positively correlated with energy allocation to the top 5 relationships in the core network (*r* = .49, *p* < .001). Conversely, energy allocation to the middle layer was negatively correlated with energy allocation to the top 5 relationship within core network (*r* =  ‒.29, *p* < .001). Energy allocation to the outer layer was strongly negatively correlated with energy allocation to the top 5 relationships within core network (*r* =  ‒.47, *p* < .001). Thus, people who assigned more energy to the inner Dunbar layer also assigned more energy to their closest individual relationships within their core networks.

### Personality as a predictor of energy allocation

RQ2a asked whether extraversion, agreeableness, conscientiousness, neuroticism, and openness would be associated with differential energy allocation across personal network layers. The results of the three broader network models (top 5: Model 1A; top 6–15: Model 1B; top 16–150: Model 1C; see Table 2) indicated that conscientiousness was associated with greater energy allocation to the inner layer (*b* = .65, *p* = .009; Model 1a), less energy allocation to the middle layer (*b* =  ‒.36, *p* = .006; Model 1b), and not associated with energy allocation to the outer layer (*b* =  ‒.28, *p* = .12; Model 1c). Extraversion, agreeableness, neuroticism, or openness were not associated with energy allocation to the three broader network layers.

**Table 2:** OLS regression models predicting energy allocation.

	*Dependent variable:*		
	Top 5	Top 6–15	Top 16–150
	(Model 1a)	(Model 1b)	(Model 1c)
Age	.002	.013	‒.015
Female	3.500^*^	.205	‒3.705^***^
Single	6.548^***^	‒.355	‒6.193^***^
Relocations	2.965^***^	‒.832^***^	‒2.134^***^
Big 5 Extraversion^1^	.335	‒.207	‒.129
Big 5 Openness	.197	‒.046	‒.150
Big 5 Conscientiousness	.646^**^	‒.362^**^	‒.284
Big 5 Agreeableness	‒.375	.295^*^	.080
Big 5 Neuroticism	.025	‒.120	.095
Self-esteem	‒1.619^**^	1.121^***^	.498
Observations	906	906	906
*R* ^2^	.112	.052	.112
Adjusted *R*^2^	.102	.041	.102

*Note:*

* *p* < 0.05;

** *p* < 0.01;

*** *p* < 0.001

^1^We ran additional analyses with each trait entered separately, and the results were not meaningfully different from the current findings.

RQ2b asked whether extraversion, agreeableness, conscientiousness, neuroticism, and openness would be associated with perceived energy allocation to relationships within core networks. The results show that none of the personality traits were associated with perceived energy allocation to the 1^st^–5^th^ relationships (Model 2a in Table 3) or 6^th^–8^th^ relationships (Model 2b in Table 3) within core networks.

**Table 3:** Energy allocation to relationships within core network.

	*Dependent variable:*	
	1^st^‒5^th^ (Model 2a)	6^th^‒8^th^ (Model 2b)
Age	‒.016	.027
Female	.752^***^	‒1.254^***^
Single	.080	‒.134
Relocations	.154^**^	‒.257^**^
Number of friends	.212^***^	‒.353^***^
Number of family	.283^***^	‒.473^***^
Number of coworkers	.207 ^*^	‒.345 ^*^
Network communities^1^	.100	‒.167
Network density^1^	‒.260^**^	.434^**^
Big 5 extraversion	.010	‒.017
Big 5 openness	.054	‒.091
Big 5 conscientiousness	.050	‒.083
Big 5 agreeableness	.006	‒.010
Big 5 neuroticism	.058	‒.096
Self-esteem	‒.173 ^*^	.288 ^*^
Observations	906	906
*R* ^2^	.091	.091
Adjusted *R*^2^	.076	.076

*Note*.

* *p* < .05;

** *p* < .01;

*** *p* < .001.

^1^Self-reported network communities and density with visual measures were used in the above models.

### Self-Esteem as a Predictor of Energy Allocation

RQ3a asked whether self-esteem would be associated with perceived energy allocation across the three broader network layers. Across the three layers, self-esteem was associated with less energy allocation to the inner layer (*b* =  ‒1.62, *p* = .003; Model 1a) and greater energy allocation to the middle layer (*b* =  1.12, *p* < .001; Model 1b), but was not associated with energy allocation to the outer layer (*b* = .50, *p* = .20; Model 1c). See Table 2.

RQ3b asked how self-esteem would be associated with perceived energy allocation to relationships within core networks. The results show that self-esteem was negatively associated with perceived energy allocation to the 1^st^–5^th^ relationships (*b* =  ‒.17, *p* = .02; Model 2a) and positively associated with perceived energy allocation to the 6^th^–8^th^ relationships within core networks (*b* = .29, *p* = .02; Model 2b). See Table 3.

## Discussion

There is wide interest across the social sciences in how people divide their attention and communication among personal relationships. Yet we know surprisingly little about how people *think about* their energy allocation across different relationships. To address this gap, this study investigated individual differences in energy allocation to a) different layers of personal networks and b) different relationships within core networks. Moreover, we explored trait-level predictors of energy allocation. Results indicated that people varied greatly in how they see their energy distribution to different layers of personal networks, as well as among their closest relationships. Contrary to our expectations, extraversion did not predict higher energy allocation to certain layers of personal networks or specific ties within core networks. However, we discovered that self-esteem was associated with less energy to the inner layer and greater energy to the middle layer. Altogether, our study paves the way for future research to examine how and why people reflect on their personal networks in distinctive ways.

Past work has shown that there are notable individual differences in how people engage with their ties [28,55]. Although plenty of studies have examined the number of relationships people maintain, here we assessed how people divide their energy between the same number of relationships—at least in their minds. In doing so, we shift the focus to *perceived* energy allocation, rather than indicators such as raw counts of communication or time spent interacting [44,96,97]. Consistent with Dunbar and colleagues’ framework, our data showed that people perceive that they devote substantially more energy toward the core/inner (vs. peripheral/outer) layers of their networks. Indeed, people appeared to have an intuitive sense or lay theory of how their social relationships are stratified across different layers.

To be sure, communication and interaction metrics can also be useful for examining the characteristics of social network structure, such as tie strength [98,99]. However, they are insufficient for capturing how individuals compartmentalize and prioritize their relationships in their heads—or how the cognitive structures reflecting those relationships are linked to distinct benefits [82]. By evaluating energy allocation to individuals’ own relationships, our study captured a more direct understanding of how people prioritize their ties in their minds. Given limited time and energy, perceived energy allocation is likely to reflect the choices people make about investing in and prioritizing certain social ties. Nevertheless, caution must be exercised when interpreting the findings as evidence for Dunbar’s Numbers, as it is possible that the observed patterns may reflect the most common characteristics of core networks today more so than a strict or universal rule about human social capacities.

Notably, we observed significant variation among our participants in perceived energy allocation 1) across personal layers and 2) within core networks. Our study thus reinforces the need to consider the intervals around Dunbar’s headline numbers. By comparing and contrasting the heterogeneity observed across the network layers, our study sheds light on the diverse ways that people see their personal networks from different vantage points. Our study thus provides insight into how people differentially perceive their relationship maintenance by quantifying energy allocation to different parts of their networks. Future work can investigate how different types of social interactions and experiences have different implications for energy allocation, as suggested by the communicate bond belong (CBB) theory [11]. In addition, follow-up work can test whether perceiving greater energy allocation to a particular part of one’s network is associated with objective indicators of relationship maintenance.

Our second set of RQs investigated the role of personality traits—and extraversion, in particular—in predicting energy allocation. This aim echoed prior research indicating links between personality traits and personal network structure and perception [55]. Yet existing work has not considered the links between personality and perceived energy allocation, particularly at the core network level. Surprisingly, we found that extraversion was not associated with energy allocation to network layers or relationships within core networks. Previous research has shown that extraverts tend to distribute their time more widely across social relationships, with their average emotional closeness to network members being lower than introverts [100,66]. Thus, the lack of association between extraversion and energy allocation raises the possibility that extroverts, compared with introverts, may exert more energy overall [101,102]—but do so in an even manner that does not change the overall distribution across layers.

By contrast, conscientiousness was found to be associated with people’s perceptions of their personal networks. Specifically, conscientiousness was found to be associated with greater focus on the inner layer (vs. outer layers). Conscientiousness is characterized by competence, motivation, and dependability [61]. Research has shown that conscientiousness is associated with high friendship quality and low relationship conflict [103]. These positive relational outcomes may have to do with our finding that they prioritize the most critical social support layer (i.e., one’s closest ties). By focusing their attention and effort on cultivating and nurturing the inner layer, conscientious individuals may help to ensure that their most significant social needs are met (at least in their minds). The flip side could also be true: those high in conscientiousness could be feel more motivated to allocating energy to the needs of others. In sum, our findings provide early insights into the potential role—or lack thereof—of personality in shaping personal network reflection through the crucial lens of energy allocation.

We also found that self-esteem was positively associated with the tendency to allocate energy to the middle layer (top 6–15)—in place of the inner layer—of personal networks. Similar findings were also observed for relationships within participants’ core networks. That is, high self-esteem was associated with less energy allocation to the top five relationships and more allocation to the remaining three relationships. In turn, our study sheds light on the commonly overlooked role of the middle network layer under Dunbar and colleagues’ framework (i.e., sympathy group). Our findings revealed that individuals with high (vs. low) self-esteem viewed this middle layer differently: they reported allocating more energy to those close (but not very close) relationships. Of note, the increased focus on the middle layer dislodged energy that would have gone to the inner layer. Existing research has primarily focused on the dichotomy between strong and weak ties [97,104,105]. Past work has paid less attention to the relationships that fall between strong and weak ties—i.e., those in the middle layer of personal networks such as prescribed by Dunbar’s “sympathy” layer (top 15 ties). As a result, our study highlights the potential of placing greater attention on the middle strata of personal networks.

Why might people who focus on “middle” ties exhibit higher self-esteem? One possibility is that people with higher self-esteem feel socially comfortable or confident enough to expand their network focus beyond their closest relationships (see [106] for hedonic flexibility principle; [107,108] for similar arguments with attachment styles and exploration). Of note, past research has linked trait social anxiety to a preference for close relationships, which offer a form of network “safety” [109]. By distributing energy to more distal relationships, higher self-esteem individuals can support a larger social network, which may allow them to accrue more social resources in aggregate over time. Alternatively, an additional explanation of our finding through the lens of sociometer theory would suggest that placing greater focus on more distal relationships (i.e., top 6–15 relationships) can make people feel good about themselves (i.e., higher self-esteem). This possibility also makes sense given that distal (or diverse) relationships can provide different types of social support, such as access to novel information or opportunities (e.g., [1,47]) that can address varying needs in life [110].

Regardless of the specific mechanisms at play, our findings reinforce the value of combining perspectives from sociometer theory [69] and Dunbar’s framework [17,22] to understand the implications of personal network perception. In considering how people distribute their social time to different network sectors in conjunction with self-esteem, we open new avenues for sociometer theory. Indeed, in line with the principles of sociometer theory, our data showed that self-esteem was strongly positively correlated with network satisfaction (see Table 1). This finding implies that self-esteem is not only reflective of energy allocation tendencies, but also linked to satisfaction with one’s social world. Hence, our study sheds light on the intricate interplay between the self-concept and perceived social networks, synthesizing sociometer theory with social network perspectives.

Although our study provides a step forward in measuring individual differences in perceived energy allocation, several limitations deserve attention. First, the cross-sectional data drawn from an online Mechanical Turk sample does not allow us to make directional or generalizable claims regarding the direct relationship between self-esteem or personality traits and energy allocation. Moreover, it is possible that self-esteem and personality traits influence other network characteristics (e.g., network density, communities), which, in turn, affect energy allocation more directly. However, the cross-sectional design of the current study limited in our capacity to test the mediating role of network density and communities. Future research can adopt longitudinal designs (e.g., [111]) to shed light on energy allocation in personal networks over time. Moreover, the sample used in this study is not representative, limiting the generalizability of the findings. For example, socioeconomic status (SES) might influence the energy allocation process or average network size [112,113]. Therefore, future research should use more representative samples to improve the robustness and applicability of the results across broader populations.

Second, our use of a fixed-size name generator to control for perceptual biases and individual differences in network size also comes with limitations. First, the design choices may have contributed to the observed differences between the 1^st^–5^th^ relationships and those listed 6^th^–8^th^. Hence, by not defining a formal threshold for what counts as a relationship, our data cannot speak to how strict definitions of core ties are perceived across layers. Additionally, it should be noted that our approach may capture a mix of both "strong" and "weak" ties for some participants; for others, it may only capture strong ties, depending on their network sizes. As a result, the personal network data collected may represent the innermost layer for some individuals, while for others, it may extend to include ties in outer layers. Nonetheless, our ultimate priority was to limit the subjectivity in naming alters—i.e., the number of people participants considered when submitting their responses. Consistent with past work [82], this approach helps control for cognitive biases that could introduce additional confounds. Still, future research should explore and clarify the relationship between energy allocation and network size.

Third, our findings may have been affected by other aspects of the study design beyond the name generation process. For example, by reading key perspectives from Dunbar’s research in the survey instructions, participants may have been cued to stratify their energy allocation accordingly. Similarly, although we asked participants to nominate specific individuals for the inner circle, we did not ask them to do the same for the middle and outer circles (given potential survey fatigue and accuracy issues). This may have led them to think more abstractly about their middle and outer circles and potentially contributed to the discrepancies in reported energy allocation between the two types of networks. Although our measures of Dunbar’s broader network layers and the core network name generator share some conceptual overlap, their differences do not allow for direct comparisons. Nonetheless, we found a strong correlation between energy allocation to the inner layer and the top 5 ties in the core network, *t*(16.91) =  904, *r* = .49, indicating a significant alignment between these two measures. Future research should further investigate the (mis)alignments between core and broader network perceptions given their theoretical relevance to each other.

Fourth, our focus on trait-level predictors of network perception does not provide insights into the precise processes guiding energy allocation in daily life. Future work can test potential mechanisms to provide deeper insights into how channeling energy to different parts of personal networks—at least in one’s mind—has implications for individuals (and their ties).

Last, the present study did not test the role of relationship role (e.g., family members vs. colleagues) in energy allocation. Future research may investigate whether Dunbar’s Numbers hold when accounting for the specific roles of alters. Specifically, adopting the network scale-up method (NSUM) would enable the estimation of the size of various relationship roles and facilitate comparisons between the layers of social networks [114]. Thus, other approaches to study design have the potential to  offer valuable insights into the dynamics of social networks,relationship roles, and energy allocation.

## Conclusion

Our exploratory study demonstrates the value of studying the mental process through which people actively think about and wholistically assess their personal networks. Participants in our study were asked to reflect on a global set of relationships at once, rather than thinking about each relationship or pair of relationships independently. Such reflection matters because people may vary considerably in their perceptions toward a wide range of aspects of their personal networks (e.g., the amount of social support)—even if their networks are similar in size and structure [82]. Furthermore, differences in how people perceive their networks can have a significant impact on their behavior [45,96]. More research is needed to study the importance of personal network reflection, especially considering the increasing range of technologies that provide options for relationship organization and curation [12]. Altogether, our findings suggest our understanding of personal networks can be advanced by probing how people *reflect* on their social energy allocation.

## References

[1] Cohen S, Janicki-Deverts D. Can we improve our physical health by altering our social networks?. Perspect Psychol Sci. 2009;4(4):375–8. doi: 10.1111/j.1745-6924.2009.01141.x 20161087 PMC2744289

[2] Cohen S, Wills TA. Stress, social support, and the buffering hypothesis. Psychol Bull. 1985;98(2):310–57. doi: 10.1037/0033-2909.98.2.310 3901065

[3] House JS, Landis KR, Umberson D. Social relationships and health. Science. 1988;241(4865):540–5. doi: 10.1126/science.3399889 3399889

[4] Hülür G, Macdonald B. Rethinking social relationships in old age: digitalization and the social lives of older adults. Am Psychol. 2020;75(4):554–66. doi: 10.1037/amp0000604 32378949

[5] Thoits PA. Stress, coping, and social support processes: where Are We? What Next?. J Health Soc Behav. 1995;35:53. doi: 10.2307/2626957 7560850

[6] Collins HK, Hagerty SF, Quoidbach J, Norton MI, Brooks AW. Relational diversity in social portfolios predicts well-being. Proc Natl Acad Sci U S A. 2022;119(43):e2120668119. doi: 10.1073/pnas.2120668119 36252003 PMC9618086

[7] Hall JA. How many hours does it take to make a friend?. J Soc Pers Relations. 2018;36(4):1278–96. doi: 10.1177/0265407518761225

[8] Fehr B. Friendship formation. In: Sprecher S, Wenzel A, Harvey J (Editors). Handbook of relationship initiation. Psychology Press. 29–54.

[9] Stiller J, Dunbar RIM. Perspective-taking and memory capacity predict social network size. Soc Netw. 2007;29(1):93–104. doi: 10.1016/j.socnet.2006.04.001

[10] Miritello G, Moro E, Lara R, Martínez-López R, Belchamber J, Roberts SG, et al. Time as a limited resource: Communication strategy in mobile phone networks. Social Networks. 2013;35(1): 89–95. doi: 10.1016/j.socnet.2013.01.003

[11] Hall JA, Davis DC. Proposing the communicate bond belong theory: evolutionary intersections with episodic interpersonal communication. Commun Theor. 2016;27(1):21–47. doi: 10.1111/comt.12106

[12] Bayer JB, Hofstra B. Toward curation and personality-driven social networks. Nature Human Behaviour. 2019;4(2): 123–125. doi: 10.1038/s41562-019-0751-y 31570759

[13] Hampton KN. Persistent and pervasive community. Am Behav Sci. 2015;60(1):101–24. doi: 10.1177/0002764215601714

[14] Wellman B. Is Dunbar’s number up?. Br J Psychol. 2012;103(2):174–6; discussion 180-2. doi: 10.1111/j.2044-8295.2011.02075.x 22506743

[15] Bayer JB, Sweitzer MD, Xiang H, Mohan S, Myers E. Reimagining the Personal Network: The Case of Path. Social Media + Society. 2022;8(3): null. doi: 10.1177/20563051221119475

[16] Dunbar RIM. The social brain hypothesis. Evol Anthropol. 1998;6(5):178–90. doi: 10.1002/(sici)1520-6505(1998)6:5<178::aid-evan5>3.0.co;2-8

[17] Dunbar RIM. Coevolution of neocortical size, group size and language in humans. Behav Brain Sci. 1993;16(4):681–94. doi: 10.1017/s0140525x00032325

[18] Barton RA, Dunbar RIM. Evolution of the social brain. Machiavellian Intelligence II. 1997:240–63. doi: 10.1017/cbo9780511525636.010

[19] Acedo-Carmona C, Gomila A. Una revisión crítica de la hipótesis del cerebro social de Dunbar. Rev int sociol. 2016;74(3):e037. doi: 10.3989/ris.2016.74.3.037

[20] Powell J, Lewis PA, Roberts N, García-Fiñana M, Dunbar RIM. Orbital prefrontal cortex volume predicts social network size: an imaging study of individual differences in humans. Proc Biol Sci. 2012;279(1736):2157–62. doi: 10.1098/rspb.2011.2574 22298855 PMC3321718

[21] Dunbar RIM. Structure and function in human and primate social networks: implications for diffusion, network stability and health. Proc Math Phys Eng Sci. 2020;476(2240):20200446. doi: 10.1098/rspa.2020.0446 32922160 PMC7482201

[22] Zhou W-X, Sornette D, Hill RA, Dunbar RIM. Discrete hierarchical organization of social group sizes. Proc Biol Sci. 2005;272(1561):439–44. doi: 10.1098/rspb.2004.2970 15734699 PMC1634986

[23] Gonçalves B, Perra N, Vespignani A. Modeling users’ activity on twitter networks: validation of Dunbar’s number. PLoS One. 2011;6(8):e22656. doi: 10.1371/journal.pone.0022656 21826200 PMC3149601

[24] Mac Carron P, Kaski K, Dunbar R. Calling Dunbar’s numbers. Soc Netw. 2016;47:151–5. doi: 10.1016/j.socnet.2016.06.003

[25] Haerter JO, Jamtveit B, Mathiesen J. Communication dynamics in finite capacity social networks. Phys Rev Lett. 2012;109(16):168701. doi: 10.1103/PhysRevLett.109.168701 23215144

[26] Dunbar R. Friends: Understanding the power of our most important relationships. 2022.

[27] Roy C, Bhattacharya K, Dunbar RIM, Kaski K. Turnover in close friendships. Sci Rep. 2022;12(1):11018. doi: 10.1038/s41598-022-15070-4 35773294 PMC9247060

[28] Dunbar RIM. The anatomy of friendship. Trends Cogn Sci. 2018;22(1):32–51. doi: 10.1016/j.tics.2017.10.004 29273112

[29] Dunbar RIM. Do online social media cut through the constraints that limit the size of offline social networks?: 9. 10.1098/rsos.150292 PMC4736918 26909163

[30] Sutcliffe A, Dunbar R, Binder J, Arrow H. Relationships and the social brain: integrating psychological and evolutionary perspectives. Br J Psychol. 2012;103(2):149–68. doi: 10.1111/j.2044-8295.2011.02061.x 22506741

[31] Tamarit I, Cuesta JA, Dunbar RIM, Sánchez A. Cognitive resource allocation determines the organization of personal networks. Proc Natl Acad Sci U S A. 2018;115(33):8316–21. doi: 10.1073/pnas.1719233115 30049707 PMC6099867

[32] Dunbar RI, Spoors M. Social networks, support cliques, and kinship. Hum Nat. 1995;6(3):273–90. doi: 10.1007/BF02734142 24203093

[33] Hampton KN, Sessions LF, Her EJ. Core networks, social isolation, and new media. Inform Commun Soc. 2011;14(1):130–55. doi: 10.1080/1369118x.2010.513417

[34] Bayer JB, O’Donnell MB, Cascio CN, Falk EB. Brain sensitivity to exclusion is associated with core network closure. Sci Rep. 2018;8(1):16037. doi: 10.1038/s41598-018-33624-3 30375417 PMC6207694

[35] Granovetter M. The strength of weak ties: a network theory revisited. Soc Theory. 1983;1:201. doi: 10.2307/202051

[36] Hill RA, Dunbar RIM. Social network size in humans. Hum Nat. 2003;14(1):53–72. doi: 10.1007/s12110-003-1016-y 26189988

[37] Roberts SGB, Dunbar RIM, Pollet TV, Kuppens T. Exploring variation in active network size: constraints and ego characteristics. Social Networks. 2009;31(2):138–46. doi: 10.1016/j.socnet.2008.12.002

[38] Flynn FJ, Reagans RE, Guillory L. Do you two know each other? Transitivity, homophily, and the need for (network) closure. J Pers Soc Psychol. 2010;99(5):855–69. doi: 10.1037/a0020961 20954787

[39] Lamkin J, Clifton A, Campbell WK, Miller JD. An examination of the perceptions of social network characteristics associated with grandiose and vulnerable narcissism. Personal Disord. 2014;5(2):137–45. doi: 10.1037/per0000024 24364501

[40] Reissman C, Aron A, Bergen MR. Shared activities and marital satisfaction: causal direction and self-expansion versus boredom. J Soc Personal Relationships. 1993;10(2):243–54. doi: 10.1177/026540759301000205

[41] Rusbult CE. A longitudinal test of the investment model: The development (and deterioration) of satisfaction and commitment in heterosexual involvements. J Personal Soc Psychol. 1983;45(1):101–17. doi: 10.1037/0022-3514.45.1.101

[42] Rusbult CE. Commitment and satisfaction in romantic associations: A test of the investment model. J Exp Soc Psychol. 1980;16(2):172–86. doi: 10.1016/0022-1031(80)90007-4

[43] Small ML, Sukhu C. Because they were there: access, deliberation, and the mobilization of networks for support. Soc Netw. 2016;47:73–84. doi: 10.1016/j.socnet.2016.05.002

[44] Perry BL, Pescosolido BA. Functional specificity in discussion networks: the influence of general and problem-specific networks on health outcomes. Soc Netw. 2010;32(4):345–57. doi: 10.1016/j.socnet.2010.06.005

[45] Bayer JB, Lewis NA, Stahl JL. Who Comes to Mind? Dynamic Construction of Social Networks. Current Directions in Psychological Science. 2020;29(3): 279–285. doi: 10.1177/0963721420915866

[46] Fitzsimons GM, Shah JY. How goal instrumentality shapes relationship evaluations. J Pers Soc Psychol. 2008;95(2):319–37. doi: 10.1037/0022-3514.95.2.319 18665705

[47] Lee DS, Fujita K. From whom do people seek what type of support? A regulatory scope perspective. J Pers Soc Psychol. 2023;124(4):796–811. doi: 10.1037/pspi0000405 35862084

[48] Taquet M, Quoidbach J, de Montjoye Y-A, Desseilles M, Gross JJ. Hedonism and the choice of everyday activities. Proc Natl Acad Sci U S A. 2016;113(35):9769–73. doi: 10.1073/pnas.1519998113 27528666 PMC5024602

[49] Tsang S, Barrentine K, Chadha S, Oishi S, Wood A. Social exploration: how and why people seek new connections. Psychol Rev. 2024:10.1037/rev0000499. doi: 10.1037/rev0000499 39264681

[50] Shaver P, Hazan C. Being lonely, falling in love. J Soc Behav Personal. 1987;2.

[51] Simpson JA. Influence of attachment styles on romantic relationships. J Personal Soc Psychol. 1990;59(5):971–80. doi: 10.1037/0022-3514.59.5.971

[52] Bowlby J. Loss, sadness and depression. New York: Basic Books; 1980.

[53] Roberts SGB, Wilson R, Fedurek P, Dunbar RIM. Individual differences and personal social network size and structure. Pers Individ Differ. 2008;44(4):954–64. doi: 10.1016/j.paid.2007.10.033

[54] Maya-Jariego I, Letina S, González Tinoco E. Personal networks and psychological attributes: exploring individual differences in personality and sense of community and their relationship to the structure of personal networks. Net Sci. 2019;8(2):168–88. doi: 10.1017/nws.2019.15

[55] Selden M, Goodie AS. Review of the effects of five factor model personality traits on network structures and perceptions of structure. Soc Netw. 2018;52:81–99. doi: 10.1016/j.socnet.2017.05.007

[56] Sprecher S. Acquaintanceships (weak ties): their role in people’s web of relationships and their formation. Pers Relationships. 2022;29(3):425–50. doi: 10.1111/pere.12430

[57] John O, Srivastava S. The big five trait taxonomy: History, measurement, and theoretical perspectives. In: Pervin LA, John OP (Editors). Handbook of personality: theory and research. New York: Guilford Press; 1999:102–38.

[58] Harari GM, Vaid SS, Müller SR, Stachl C, Marrero Z, Schoedel R, et al. Personality sensing for theory development and assessment in the digital age. Eur J Pers. 2020;34(5):649–69. doi: 10.1002/per.2273

[59] Centellegher S, López E, Saramäki J, Lepri B. Personality traits and ego-network dynamics. PLoS One. 2017;12(3):e0173110. doi: 10.1371/journal.pone.0173110 28253333 PMC5333865

[60] Alshamsi A, Pianesi F, Lepri B, Pentland A, Rahwan I. Network diversity and affect dynamics: the role of personality traits. PLoS One. 2016;11(4):e0152358. doi: 10.1371/journal.pone.0152358 27035904 PMC4817969

[61] Costa PT Jr, McCrae RR. Four ways five factors are basic. Pers Individ Differ. 1992;13(6):653–65. doi: 10.1016/0191-8869(92)90236-i

[62] Digman JM. Personality structure: emergence of the five-factor model. Annu Rev Psychol. 1990;41(1):417–40. doi: 10.1146/annurev.ps.41.020190.002221

[63] Smółka P, Szulawski M. personality traits and motivational traits as predictors of social competence. implication for occupational selection process. EEIM. 2011;22(4):111–26. doi: 10.5604/01.3001.0009.5542

[64] Bolger N, Eckenrode J. Social relationships, personality, and anxiety during a major stressful event. J Pers Soc Psychol. 1991;61(3):440–9. doi: 10.1037//0022-3514.61.3.440 1941515

[65] Kalish Y, Robins G. Psychological predispositions and network structure:the relationship between individual predispositions, structural holes and network closure. Soc Netw. 2006;28(1):56–84. doi: 10.1016/j.socnet.2005.04.004

[66] Pollet TV, Roberts SGB, Dunbar RIM. Extraverts have larger social network layers. J Individ Differ. 2011;32(3):161–9. doi: 10.1027/1614-0001/a000048

[67] Antonoplis S, John OP. Who has different-race friends, and does it depend on context? Openness (to other), but not agreeableness, predicts lower racial homophily in friendship networks. J Pers Soc Psychol. 2022;122(5):894–919. doi: 10.1037/pspp0000413 35404642

[68] Porter CM, Rigby JR. Relationship context and personality shape people’s preferences for network relationship partners. Pers Relationships. 2019;26(2):310–30. doi: 10.1111/pere.12275

[69] Leary MR, Baumeister RF. The nature and function of self-esteem: Sociometer theory. Adv Exp Soc Psychol. 2000:1–62. doi: 10.1016/s0065-2601(00)80003-9

[70] Leary MR. Sociometer theory and the pursuit of relational value: Getting to the root of self-esteem. Eur Rev Soc Psychol. 2005;16(1):75–111. doi: 10.1080/10463280540000007

[71] Baumeister RF, Campbell JD, Krueger JI, Vohs KD. Does high self-esteem cause better performance, interpersonal success, happiness, or healthier lifestyles?. Psychol Sci Public Interest. 2003;4(1):1–44. doi: 10.1111/1529-1006.01431 26151640

[72] Coopersmith S. Antecedents of self-esteem. New York, NY: W.H. Freeman; 1968.

[73] Xu S, Li W, Zhang W. The dynamics of social capital: examining the reciprocity between network features and social support. J Comput Med Commun. 2021;26(6):362–83. doi: 10.1093/jcmc/zmab014

[74] Anthony DB, Wood JV, Holmes JG. Testing sociometer theory: Self-esteem and the importance of acceptance for social decision-making. J Exp Soc Psychol. 2007;43(3):425–32. doi: 10.1016/j.jesp.2006.03.002

[75] Cameron JJ, Granger S. Does self-esteem have an interpersonal imprint beyond self-reports? a meta-analysis of self-esteem and objective interpersonal indicators. Pers Soc Psychol Rev. 2019;23(1):73–102. doi: 10.1177/1088868318756532 29482451

[76] Feeney BC, Collins NL. A new look at social support: a theoretical perspective on thriving through relationships. Pers Soc Psychol Rev. 2015;19(2):113–47. doi: 10.1177/1088868314544222 25125368 PMC5480897

[77] Feeney BC. A secure base: responsive support of goal strivings and exploration in adult intimate relationships. J Pers Soc Psychol. 2004;87(5):631–48. doi: 10.1037/0022-3514.87.5.631 15535776

[78] Lee DS, Ybarra O, Gonzalez R, Ellsworth P. I-Through-we: how supportive social relationships facilitate personal growth. Pers Soc Psychol Bull. 2018;44(1):37–48. doi: 10.1177/0146167217730371 28918683

[79] Wilcox K, Stephen AT. Are close friends the enemy? online social networks, self-esteem, and self-control. J Consum Res. 2012;40(1):90–103. doi: 10.1086/668794

[80] Smith EB, Brands RA, Brashears ME, Kleinbaum AM. Social networks and cognition. Annu Rev Sociol. 2020;46(1):159–74. doi: 10.1146/annurev-soc-121919-054736

[81] Marcum CS, Lienert J, Goldring M, Lin J, Miggins A, Moss ME, et al. Ego-centered cognitive social structures of close personal networks in the United States. Open Sci Framework. 2022. doi: 10.17605/OSF.IO/4UGK6

[82] Lee DS, Stahl JL, Bayer JB. Social resources as cognitive structures: thinking about a dense support network increases perceived support. Soc Psychol Q. 2020;83(4):405–22. doi: 10.1177/0190272520939506

[83] Krackhardt D. Cognitive social structures. Social Networks. 2002;9(2): 109–134. doi: 10.1016/0378-8733(87)90009-8

[84] Brands RA. Cognitive social structures in social network research: A review. Journal of Organizational Behavior. 2013;34(S1): null. doi: 10.1002/job.1890

[85] Hlebec V, Kogovšek T. How (not) to measure social support networks. Adv Meth Stat. 2011;8(2) doi: 10.51936/nxzu1540

[86] Maya Jariego I. Why name generators with a fixed number of alters may be a pragmatic option for personal network analysis. Am J Community Psychol. 2018;62(1–2):233–8. doi: 10.1002/ajcp.12271 30216459

[87] Kogovšek T, Hlebec V. Effects of limitation of number of alters and time frame in the Burt name generator. Adv Meth Stat. 2005;2(1):. doi: 10.51936/xmwm9068

[88] Casciaro T. Seeing things clearly: social structure, personality, and accuracy in social network perception. Soc Netw. 1998;20(4):331–51. doi: 10.1016/s0378-8733(98)00008-2

[89] Mehra A, Borgatti SP, Soltis S, Floyd T, Halgin DS, Ofem B, et al. Imaginary worlds: using visual network scales to capture perceptions of social networks. Res Sociol Organ. 2014:315–36. doi: 10.1108/s0733-558x(2014)0000040016

[90] Rammstedt B, John OP. Measuring personality in one minute or less: a 10-item short version of the big five inventory in English and German. J Res Pers. 2007;41(1):203–12. doi: 10.1016/j.jrp.2006.02.001

[91] Robins RW, Hendin HM, Trzesniewski KH. Measuring global self-esteem: construct validation of a single-item measure and the rosenberg self-esteem scale. Pers Soc Psychol Bull. 2001;27(2):151–61. doi: 10.1177/0146167201272002

[92] Bhattacharya K, Ghosh A, Monsivais D, Dunbar RIM, Kaski K. Sex differences in social focus across the life cycle in humans. R Soc Open Sci. 2016;3(4):160097. doi: 10.1098/rsos.160097 27152223 PMC4852646

[93] Burton-Chellew MN, Dunbar RIM. Romance and reproduction are socially costly. Evol Behav Sci. 2015;9(4):229–41. doi: 10.1037/ebs0000046

[94] Roberts SBG, Dunbar RIM. Managing relationship decay : network, gender, and contextual effects. Hum Nat. 2015;26(4):426–50. doi: 10.1007/s12110-015-9242-7 26489745 PMC4626528

[95] Kloke JD, McKean JW. Rfit: rank-based estimation for linear models. R J. 2012;4(1):57.

[96] Lai C-H. Motivations, usage, and perceived social networks within and beyond social media. J Comput Med Commun. 2019;24(3):126–45. doi: 10.1093/jcmc/zmz004

[97] Small ML. Weak ties and the core discussion network: Why people regularly discuss important matters with unimportant alters. Soc Netw. 2013;35(3):470–83. doi: 10.1016/j.socnet.2013.05.004

[98] Jones JJ, Lambiotte R, Settle JE, Bond RM, Fariss CJ, Marlow C, et al. Inferring Tie Strength from Online Directed Behavior. PLoS ONE. 2013;8(1): e52168. doi: 10.1371/journal.pone.0052168 23300964 PMC3534669

[99] Burke M, Kraut RE. The relationship between facebook use and well-being depends on communication type and tie strength. J Comput-Mediat Comm. 2016;21(4):265–81. doi: 10.1111/jcc4.12162

[100] Bodun DS, Omoboyowa DA, Olofinlade VF, Ayodeji AO, Mauri A, Ogbodo UC, et al. In-silico-based lead optimization of hit compounds targeting mitotic kinesin Eg5 for cancer management. In Silico Pharmacol. 2025;13(1):9. doi: 10.1007/s40203-024-00300-6 39780769 PMC11703796

[101] Wilt J, Revelle W. Handbook of individual differences in social behavior. Handbook of individual differences in social behavior. New York: Guilford Press; 2009;27–45 p.

[102] Tov W, Nai ZL, Lee HW. Extraversion and agreeableness: divergent routes to daily satisfaction with social relationships. J Pers. 2016;84(1):121–34. doi: 10.1111/jopy.12146 25345667

[103] Demır M, Weitekamp LA. I am so happy ’cause today i found my friend: friendship and personality as predictors of happiness. J Happiness Stud. 2006;8(2):181–211. doi: 10.1007/s10902-006-9012-7

[104] Elmer T, Boda Z, Stadtfeld C. The co-evolution of emotional well-being with weak and strong friendship ties. Net Sci. 2017;5(3):278–307. doi: 10.1017/nws.2017.20

[105] Eng N, Sun Y, Myrick JG. Who is your fitspiration? an exploration of strong and weak ties with emotions, body satisfaction, and the theory of planned behavior. Health Commun. 2023;38(7):1477–89. doi: 10.1080/10410236.2021.2012978 35001776

[106] Quoidbach J, Taquet M, Desseilles M, de Montjoye Y-A, Gross JJ. Happiness and social behavior. Psychol Sci. 2019;30(8):1111–22. doi: 10.1177/0956797619849666 31268832

[107] Feeney BC, Collins NL. The importance of relational support for attachment and exploration needs. Curr Opin Psychol. 2019;25:182–6. doi: 10.1016/j.copsyc.2018.11.011 30611023 PMC6598708

[108] Elliot AJ, Reis HT. Attachment and exploration in adulthood. J Pers Soc Psychol. 2003;85(2):317–31. doi: 10.1037/0022-3514.85.2.317 12916573

[109] Bayer JB, Triệu P, Ellison N, Schoenebeck SY, Falk EB. Rejection sensitivity and interaction quality in everyday life. J Soc Pers Relationships. 2021;38(12):3646–68. doi: 10.1177/02654075211034237

[110] Moreton J, Kelly CS, Sandstrom GM. Social support from weak ties: Insight from the literature on minimal social interactions. Soc Pers Psych. 2023;17(3): doi: 10.1111/spc3.12729

[111] González-Casado MA, Gonzales G, Molina JL, Sánchez A. Towards a general method to classify personal network structures. Soc Netw. 2024;78:265–78. doi: 10.1016/j.socnet.2024.03.004

[112] Ajrouch KJ, Blandon AY, Antonucci TC. Social networks among men and women: the effects of age and socioeconomic status. J Gerontol B Psychol Sci Soc Sci. 2005;60(6):S311–7. doi: 10.1093/geronb/60.6.s311 16260713

[113] Brooks B, Welser HT, Hogan B, Titsworth S. Socioeconomic status updates. Inf Commun Soc. 2011;14(4):529–49. doi: 10.1080/1369118x.2011.562221

[114] Laga I, Bao L, Niu X. Thirty years of the network scale-up method. J Am Stat Assoc. 2021;116(535):1548–59. doi: 10.1080/01621459.2021.1935267 37994314 PMC10665021

